# Depression as a Risk Factor for Dementia and Alzheimer’s Disease

**DOI:** 10.3390/biomedicines8110457

**Published:** 2020-10-28

**Authors:** Vanesa Cantón-Habas, Manuel Rich-Ruiz, Manuel Romero-Saldaña, Maria del Pilar Carrera-González

**Affiliations:** 1 Maimónides Institute for Biomedical Research (IMIBIC), University of Córdoba, Reina Sofia University Hospital, 14004 Córdoba, Spain; n92cahav@uco.es (V.C.-H.); z92rosam@uco.es (M.R.-S.); pcarrera@uco.es (M.d.P.C.-G.); 2 Ciber Fragility and Healthy Aging (CIBERFES), 28001 Madrid, Spain; 3 Experimental and Clinical Physiopathology Research Group, Department of Health Sciences, Faculty of Experimental and Health Sciences, University of Jaén, E-23071 Jaén, Spain

**Keywords:** dementia, Alzheimer’s disease, depression, diabetes mellitus, type 2, dyslipidemias, hypertension

## Abstract

Preventing the onset of dementia and Alzheimer’s disease (AD), improving the diagnosis, and slowing the progression of these diseases remain a challenge. The aim of this study was to elucidate the association between depression and dementia/AD and to identify possible relationships between these diseases and different sociodemographic and clinical features. In this regard, a case-control study was conducted in Spain in 2018–2019. The definition of a case was: A person ≥ 65 years old with dementia and/or AD and a score of 5–7 on the Global Deterioration Scale (GDS). The sample consisted of 125 controls; among the cases, 96 had dementia and 74 had AD. The predictor variables were depression, dyslipidemia, type 2 diabetes mellitus, and hypertension. The results showed that depression, diabetes mellitus, and older age were associated with an increased likelihood of developing AD, with an Odds Ratio (OR) of 12.9 (95% confidence interval (CI): 4.3–39.9), 2.8 (95% CI: 1.1–7.1) and 1.15 (95% CI: 1.1–1.2), respectively. Those subjects with treated dyslipidemia were less likely to develop AD (OR 0.47, 95% CI: 0.22–1.1). Therefore, depression and diabetes mellitus increase the risk of dementia, whereas treated dyslipidemia has been shown to reduce this risk.

## 1. Introduction

In recent decades, population aging has led to an increase in the number of people affected by cognitive impairment, this becoming a major problem both clinically and socially, especially in Western countries [1,2]. In this sense, while in 2016 there were 47.5 million people diagnosed with dementia, in 2050 it is expected to affect more than 135.5 million individuals [3], with Alzheimer’s disease (AD) and vascular dementia (VD) the most common form of dementia [2,4,5].

However, the big problem with these diseases is not their frequency nor their growth forecasting, but their late diagnosis. The lack of differentiation between normal processes of cognitive impairment and disease states, coupled with the characteristic features of dementia—with an appearance that tends to be insidious and a development that tends to extend over time [6]—makes the first symptoms of dementia and AD easily go unnoticed [7,8]. Therefore, patients suffering from these diseases are diagnosed too late, when neurological symptoms are actually noticeable and the disease is already in very advanced stages [9]. In fact, previous studies point to how AD probably begins decades before the first symptoms appear [10]. As a result, the identification of risk/prodromal factors becomes essential to prevent the disease and, especially, to prevent late detection/diagnosis of the disease.

The association between this disease and many risk factors has been described. Some of them, such as dyslipidemia, hypertension, or tobacco use, have clear pathophysiological mechanisms because of their vascular component [11,12]. Nevertheless, this would not be the only association between dyslipidemia and AD since recent animal studies have shown how hypercholesterolemia may favor β-amyloid deposits characteristic of AD and therefore be related to neuroinflammation and loss of neuronal function [13]. However, despite being considered a risk factor for the development of AD, the pharmacological approach to this cardiovascular risk factor in older people would not prevent the onset of AD nor slow the course of neurodegenerative disease. This leads us to question the link described above between dyslipidemia and AD [14,15].

Other risk factors, such as type 2 diabetes mellitus (T2DM), seem to have a bidirectional relationship with AD, because it involves modifications in vascular function and structure, glucose metabolism, and insulin signaling, thus contributing to neurodegeneration [16,17,18]. In fact, both dementia and T2DM share symptoms, such as inflammation and altered insulin signaling mechanisms [19]. However, the relationship between the metabolism of tau and β-amyloid proteins has not yet been elucidated [20], so while some authors focus their research on understanding the link between this accumulation and the existence of other factors, such as amylin (protein co-secreted with insulin), others analyze whether the accumulation may be part of the diabetic phenotype [21,22].

Finally, others, such as anxiety and depression seem to be related to the appearance of dementia. However, while recent studies have described anxiety as a risk factor of AD, increasing the risk of this neurodegenerative disease by up to 50% [23], the exact link between depression and dementia is unknown and controversial, to the point of not knowing if depression is associated with a future development of AD and if it could be considered a risk factor for AD, or if, on the contrary, depression is a consequence of AD [24,25,26]. It appears that depressive symptoms in older adults with cognitive impairment may be related to the distinctive amyloid and tau signs of AD [27,28], thus establishing an association between depressive symptoms and cognitive impairment in older adults [29].

In this sense, the prevailing depressive symptoms among older adults could be considered modifiers of cognitive performance, but they could also be clinical indicators or early clinical signs and symptoms of AD [30,31], which would justify a comprehensive management of neuropsychological functioning in older people diagnosed with depression, regardless of the age of onset and the disease pattern (self-limiting, incident or persistent).

In this way, prevention of the onset of dementia would involve, first, assessing and adequately diagnosing this mental disorder and, second, addressing it with appropriate pharmacological and non-pharmacological treatment [32,33].

In this context, the aim of this study was to elucidate the association between depression and dementia/AD and to identify possible relationships between these diseases and different sociodemographic and clinical features.

## 2. Material and Methods

### 2.1. Study Design

This is a case-control study developed in four nursing homes and one dementia-specific facility in each of two urban areas of southern Spain between May 2018 and October 2019.

### 2.2. Participants and Selection Criteria

The study included a control group, consisting of 125 people without dementia, and a case group consisting of 96 participants with dementia, of whom 74 subjects had AD. This allowed a double epidemiological analysis to be carried out: firstly, between the control group (*n* = 125) and the dementia case group (*n* = 96); and secondly, between the control group and the AD case group (*n* = 74).

To ensure that patients actually had dementia or AD, strict criteria, including severity levels, were used. The inclusion criteria for participants with dementia were the following:

- Age ≥ 65 years.
- Medical diagnosis of dementia or AD with a global deterioration scale (GDS) score between 5 and 7 [34]. Patients received diagnosis of dementia if they met DSM-V clinical criteria and received a diagnosis of probable or possible AD according to NINCDS/ADRDA (National Institute of Neurological and Communicative Disorders and Stroke/Alzheimer’s Disease and Related Disorders Association) criteria.
- Being included, at least, for three months in the listings of the dementia process. In the case of the institution dedicated to the care of patients with Alzheimer’s disease, patients who had used this service for at least three months were included.
- Being unable to communicate verbally.
- Having a relative or legal representative that could sign the informed consent for the participation of the patient in the study.

People with AD who had comorbidity with other major clinical neurological illness were excluded.

The inclusion criteria for participants without dementia (controls) were the following:

- Age ≥ 65 years.
- Being able to sign the informed consent for their participation in the study.
- Not presenting with a diagnosis of dementia or AD.

The recruitment of the individuals with dementia was conducted consecutively by the interventional nurses among the subjects they care for in their health care institution (lists of patients). The recruitment included all individuals (prevalent and incident cases) found during the study time in the mentioned settings. The controls were recruited, simultaneously, in the close environment of participants with dementia and/or in the area of influence of the centers involved in the recruitment of participants with dementia, among those within the same age range and who were willing to participate in this study.

The sample size was calculated for a 10% difference in the proportion of subjects presenting with diabetes mellitus (and 15% and 30% in subjects presenting with dyslipidemia and depression [35], respectively) in the group of subjects with and without dementia (calculated after a pilot study). An alpha risk of 5% and a beta risk of 20%, with a ratio of 1:1 and an estimated loss to follow-up of 10% were considered. The total number of subjects required was 219.

### 2.3. Study Measures

The main dependent variables were the diagnosis of dementia/AD and the scores of the GDS scale, whereas the main predictor variables were four chronic conditions related to dementia in previous studies: medical diagnosis of depression according to DSM-V, dyslipidemia, T2DM, and hypertension.

In addition, other study variables, such as sociodemographic characteristics (sex, age, and place of residence –rural or urban), were collected from the clinical record. Furthermore, the level of autonomy in basic activities of daily living was measured using the Barthel index. In this sense, the scales (GDS and Barthel Index) were administered by the research team at the time of data collection or, if collected from the medical record, were not more than three months old. The predictor variables (depression, dyslipidemia, T2DM, and hypertension) and the date of diagnosis of each of them were collected from the medical record to ensure the antecedents of these conditions.

### 2.4. Statistical Analysis

IBM SPSS Statistics 22.0 (SPSS/IBM, Chicago, IL, USA) and Epidat version 4.1 (Department of Sanida, Xunta de Galicia, Galicia, Spain) software was used for statistical and epidemiological treatment of the data.

Continuous variables were expressed as the mean ± standard deviation, while categorical variables were expressed as the frequency and proportion distribution. The Kolmogorov–Smirnov test with Lilliefors correction and graphical representation tests, such as P–P and Q–Q plots, was applied to test the goodness of fit to a normal distribution of the data.

A Student’s *t* test was used for variables with a normal distribution (using the Levene test for variance equality), whereas non-parametric tests, such as the U Mann–Whitney test (independent samples), were used for variables showing a non-normal distribution and were used for bivariate analysis. The Z test, chi squared test, and Fisher’s exact test were used whenever necessary for each contingency tables of categorical variables.

Bivariate and multivariate analyses were performed by binary logistic regression. Goodness-of-fit tests for the model (2 loglikelihood, goodness-of-fit statistics, Nagelkerke R2, and Hosmer-Lemeshow test) were calculated to assess the global fit of the model. Exponentiation was used for the b-coefficients in the regression models to estimate the OR, and the standard error of the b-coefficients was used to calculate the 95% confidence interval (CI). The confounding effect was analyzed for those variables of the final model whose statistical significance value was between 0.05 and 0.2. It was considered a confounding effect when the crude and adjusted Beta coefficient variation was above 10%.

Receiver Operator Characteristic (ROC) curves were constructed, and the Area Under the Curve (AUC) was calculated to determine which explanatory variables best predicted the onset of dementia and AD. The diagnostic accuracy indicators, such as sensitivity, specificity, predictive values, and Youden and Validity Indices were analyzed.

The level of statistical significance was set at *p* < 0.05, and the confidence intervals were calculated at a 95% level.

### 2.5. Limitations

Firstly, it should be noted that no estimation of the incidence or prevalence of the events of interest (depression and dementia) could be performed because this study lacked a population base.

In addition, the design type involved constraints in establishing the temporary sequence of possible exposures (depression and others) and effects (dementia and AD, with GDS 5–7), since the onset (diagnosis) of chronic conditions/diseases is always uncertain.

By including prevalent cases (in addition to incident cases), we may have included the most surviving cases, so long-term cases may have been overrepresented (“Neyman fallacy”).

For institutionalized older people, one type of selection bias, “Berkson’s bias,” should be considered, because a selection of cases from a nursing home or dementia-specific facility population could contain a higher proportion of older people with a secondary disease.

However, as an analytical observational study, the main limitation of this study is the possibility of confounding factors not covered by the design. However, efforts have been made to reduce the influence of these confounding factors by using multivariate data analysis techniques.

### 2.6. Ethical Aspects

The study was conducted in accordance with the precepts included in the Belmont report and the Helsinki Declaration (updated at the Seoul Assembly in 2008) for biomedical research. All candidates for entry into the study were informed through a Patient Information Sheet (PIS). Written informed consents were voluntarily signed by the patients. All participants were allowed to leave the study at any time. Subject anonymity and data confidentiality were guaranteed at all times. Finally, the study had the authorization of all participating centers and the permission of the Ethics Committee for Research of Andalusia (Acta nº 271, ref. 3672, approved on 5 December 2017).

## 3. Results

### 3.1. Sample Characteristics

A total of 221 participants were studied, of whom 168 (76%) were women. The overall mean age was 79.1 (8.6) years, 95% CI (78–80.3), and the range was 65–100 years. No significant differences in age between women and men were found.

A total of 48% of the sample were married, and 50% of the group of women were widows. With regard to institutionalization, 107 persons were admitted to one of two types of geriatric centers (48.4%), finding no sex differences. The sample consisted of 167 subjects living in an urban area (75.6%) compared with those living in a rural area.

A total of 96 participants had a diagnosis of dementia, 43.4% with a 95% CI (36.7–50.2) of the study subjects. Prevalence in women was 45.2% compared to 37.7% in men (*p* = 0.42). For AD (*n* = 74), the overall prevalence in the sample was 33.5% with a 95% CI (27–39.9), higher in women (35.7%) than in men (26.4%) *p* = 0.28. Finally, 22 participants were diagnosed with non-AD dementia.

With regard to the level of independence for performing basic daily life activities, the median Barthel Index score for people with dementia was 10 (max = 80 and min = 0). Consequently, 61.49% showed a total dependency, 31.25% showed a severe dependence, and 7.29% showed a moderate dependence.

Finally, the prevalence of other relevant clinical entities was: Depression (17.6%), T2DM (18.1%), hypertension (61.5%). In terms of dyslipidemia, the prevalence was 38.7%, and 74.4% of them were under treatment with statins. Table 1 shows the characteristics of the sample according to the study variables and sex.

### 3.2. Variables Associated with Dementia and AD

A double comparative analysis was carried out between the control group versus the group of cases with dementia and the group of cases with AD, by means of crude (unadjusted) binary logistic regression analysis, in order to know which study variables were associated with suffering from dementia in general, or AD in particular (Table 2).

Based on the results of Table 2, multivariate models were performed by binary logistic regression, for both dementia and Alzheimer’s disease, including those independent variables with a statistical significance of *p* ≤ 0.2 (all variables except sex and hypertension), and considering the parsimony of modeling (no more than five independent variables). The multivariate models performed were as follows:

- Model 1: Adjusted for age and depression.
- Model 2: Adjusted for age and dyslipidemias.
- Model 3: Adjusted for age, depression, dyslipidemias, and T2DM.

With regard to dementia, Table 3 shows the adjusted OR results for each explanatory variable in each of the proposed models. Table 3 shows that the age variable was involved in the three models with a high statistical significance (*p* < 0.001), as well as a stable adjusted OR value of approximately 1.16. That is, under equally adjusted variables, for each 1-year increase in the person’s age, the risk of presenting dementia increases by 16%.

The variable depression was included in models 1 and 3, and it was significantly associated with the presence of dementia in both models, its adjusted OR ranging between 13.6 (4.8–38.7) in Model 1 and 15.6 (5.3–45) in Model 3. This means that under equal variables included in the model, those subjects who suffered an episode of depression presented between 13.6- and 15.6-fold higher prevalence of dementia than subjects without depression.

The behavior of the dyslipidemia variable was not included in Model 2 (*p* = 0.12 and non-confounding effect), but was included in Model 3, obtaining an adjusted OR of 0.54 (0.27–1.1) *p* = 0.089, considering it as a confounding variable by causing a variation of the crude-adjusted value of the beta coefficient of the T2DM variable of 12.5%. This means that, under equal variables included in the model, those subjects with dyslipidemia have had a 1.85-fold (1/0.54) lower prevalence of dementia than subjects without dyslipidemia.

Finally, T2DM was associated with dementia, adjusted OR= 2.6 (1.05–6.3) *p* < 0.05. That is, under the equal variables of Model 3, subjects with diabetes suffered a 2.6-fold higher prevalence of dementia than subjects without T2DM.

Regarding AD as a dependent variable (Table 3), the results obtained were similar to those found in the multivariate models for dementia (partly explained by the collinearity between dementia-AD, being the latter part of dementia). Variables significantly associated with the outcome variables, as well as the sign and adjusted OR values, were very similar in dementia and AD models. The dyslipidemia variable was maintained in Models 2 and 3 due to its small alpha error (*p* = 0.07 and 0.056, respectively).

### 3.3. Diagnostic Accuracy of Dementia and AD

Table 4 shows the accuracy (diagnostic accuracy) and the safety and validity indicators of the multivariate logistic regression models for dementia and AD. For both variables, Model 3 adjusted for age, depression, dyslipidemia, and T2DM showed the highest goodness of fit (Nagelkerke r^2^ = 0.48), i.e., the variables included accounted for 48% of the value of the result variable.

**Figure 1:**
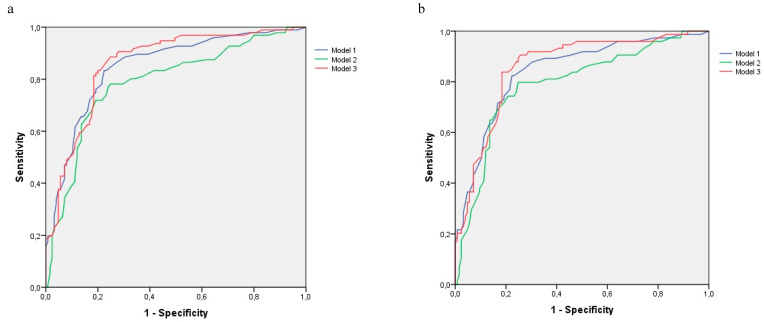
ROC curves for multivariate logistic regression models for dementia and AD outcome variables. (**a**). Outcome Variable: Dementia; (**b**). Outcome Variable: AD.

Model 3 achieved for dementia an area under the curve of 86% (ROC curves in Figure 1), a sensitivity of 77.1%, and a specificity of 81.6%, which resulted in a Youden index of 0.59, with a positive predictive value (PPV) of 76.3% and a negative predictive value (NPV) of 82.3%. The percentage of correctly classified persons (validity index) was 79.6%.

Model 3 obtained an area under the curve of 85.8% and a diagnostic validity index of 76.9% for AD. Sensitivity was 66.2%, and specificity was 83.2%, with a Youden index of 0.48. PPV and NPV were 70% and 80.6%, respectively.

## 4. Discussion

Recent research, consistent with the results of the present study, indicates that dementia and depression are diseases with a high prevalence and with a remarkable overlap in their epidemiological data [36,37].

However, the temporary sequence of this association is controversial. On the one hand, several authors point out that the presence of late depressive symptoms in older people could be the first manifestation of dementia, so depression, in this case, would be a prodromal factor of dementia [38,39]. On the other hand, some authors state that people with depression have an increased risk of being diagnosed with dementia and/or AD in old ages [40,41], results that are consistent with the present study, where those subjects with a diagnosis of depression had between 13.6 and 15.6 times higher prevalence of dementia than subjects without depression.

Nevertheless, this association between the two diseases, based on the existing literature, will be determined by the severity of the depressive symptoms, the recurrence of episodes, the general state of health of the person, and the presence of depression in adulthood [42,43,44,45].

With regard to the latter point, multiple studies indicate that the link between the two diseases would be limited by the time of onset of depression [46,47]. Therefore, age must be understood as a factor of great importance in this relationship, even more when thanks to the development of imaging techniques, it has been possible to compare the causal relationship between these two clinical entities from the pathophysiological point of view, because in people diagnosed with depression at an old age, the presence of β-amyloid plaques and accumulation of tau protein in the brain years before the presentation of dementia have been verified [48,49].

Thus, the onset of these symptoms in old ages can be understood as a prodromal factor, and in turn, the appearance of early depression can be understood as a risk factor for developing dementia and/or AD in both early and old ages [50,51].

In addition, the consideration of depressive symptoms as a prodromal factor would be higher in people diagnosed with dementia with Lewy bodies or VD than in those affected by AD [52,53].

Regarding the pathophysiological link between the two diseases, it appears to be centered on microglia activation as the basis of the process of cerebral neuroinflammation described in both diseases [54]. In this sense, the studies developed by Gathel et al., (2019) take on special relevance, because their objectives were to try to establish a relationship between depressive symptoms, cognition, and cortical amyloid in community-dwelling older adults [29].

Despite the evident relationship between these diseases, health professionals often treat these two diseases independently, focusing the treatment of dementia, particularly in the case of AD, on memory decline and forgetting to include the care of the depressive behavioral symptoms that these patients present as a key element [55]. This inadequate approach to depression in people with dementia increases functional and cognitive impairment, especially if the person also suffers from anxiety as it seems to accentuate the cognitive decline [56], thus intensifying the loss of independence of the person, and ultimately it is associated with an increase in the institutionalization of these patients [57].

Moreover, beyond depression, and as our results show, older age is a risk factor itself for developing dementia and/or AD [58,59]. If the other variables are equal, for every 1-year increase in the person’s age, the risk of developing dementia increases by 16%.

Another of the diseases described in the scientific literature due to its potential relationship with the development of dementia, and as has been proven in the results of the present study, is T2DM, which must be understood as a risk factor for dementia [60]. Specifically, the findings of the present study estimate that those with T2DM experienced 2.6 times higher prevalence of dementia than subjects without T2DM. However, this decrease in cognitive functions, particularly memory and reasoning, and therefore the development of dementia seems to depend, according to the existing literature, on two factors in people with TMD2: The duration of the disease and the glycemic control [61].

In addition, T2DM has also been defined as a risk factor specifically for patients with AD with depression, because increased serum levels of glycosylated hemoglobin favor a worsening of depressive symptoms in patients with AD. This is why adequate control of T2DM, through hygienic-dietary measures and appropriate pharmacological treatment, can reduce the severity of depression in patients with this neurodegenerative disease [62].

With regard to the influence of dyslipidemia, and in accordance with our results, it should be noted that low levels of HDL increase the risk of AD [63], as well as a greater progression of the disease [64], whereas high serum HDL levels are associated with improved memory function [65]. In this sense, according to Ward et al. (2010), there is a positive relationship between HDL levels and gray matter volume in the temporal area, and consequently with de cognitive function [66], with this relationship limited by the fact that HDL contains apolipoprotein E (APOE) [67]. In addition, recent studies indicate that increased serum LDL levels are associated with higher deposits of β-amyloid protein in the brain, resulting in increased risk of developing AD [64,68].

However, in addition to the described metabolic relationship between APOE, HDL, and AD, brain neuroinflammation is a fundamental connection point between dyslipidemia and dementia [69]. Thus, the pharmacological approach to dyslipidemia using drugs, such as statins, could not only reduce serum lipid levels, a widely recognized function of this treatment, but would help to mitigate the inflammatory process, thus benefiting all patients with AD, although the progression of this positive effect over time is still unknown [70,71].

In this regard, some studies suggest that treatment of dyslipidemia with statins decreases the risk of dementia and AD [72,73], which would support the results of the present study. Similarly, the use of statins is associated with a decrease in the incidence of all types of dementia, except VD [74]. However, it is not recommended to systematically use statin therapy in all people with dyslipidemia because of its controversial interference with cognitive function [75,76].

Health professionals will be responsible for discerning which subjects may benefit from the dual function of this pharmacological treatment because they present other risk factors contributing to the development of dementia or because they already have cognitive impairment [77,78].

## 5. Conclusions

Depression should be considered a risk factor for dementia, and especially AD. Moreover, people with diabetes are at higher risk of dementia than those without this chronic condition, so the appropriate approach to diabetes could significantly decrease this causal relationship. In addition, properly treated dyslipidemia may reduce the risk of dementia.

Regarding sociodemographic variables, age appears to be a decisive risk factor, even more relevant than depression.

## Figures and Tables

**Table 1:** Description of the sample according to sex.

Variable	Total *n* = 221	Women *n* = 168	Men *n* = 53	*p* Value
**Age**	79.1 (8.6)	79.1 (8.8)	79.1 (7.9)	0.99
**Study Group**				
**Cases**	96 (43.4%)	76 (45.2%)	20 (37.7%)	0.33
**Controls**	125 (56.6%)	92 (54.8%)	33 (62.3%)	
**Marital Status**				
**Single**	11 (5%)	9 (5.4%)	2 (3.8%)	<0.05
**Married**	106 (48%)	71 (42.3%)	35 (66%)	
**Widowed**	99 (44.8%)	84 (50%)	15 (28.3%)	
**Divorced**	5 (2.3%)	4 (2.4%)	1 (1.9%)	
**Origin of Participants**				
**Health Center**	114 (51.6%)	84 (50%)	30 (56.6%)	0.4
**Nursing Home**	107 (48.4%)	84 (50%)	23 (43.4%)	
**Living area of the Sample**				
**Urban**	167 (75.6%)	124 (73.8%)	43 (81.1%)	0.28
**Rural**	54 (24.4%)	44 (26.2%)	10 (18.9%)	
**Clinical Variables**				
**Dementia**	96 (43.4%)	76 (45.2%)	20 (37.7%)	0.42
**Alzheimer’s Disease**	74 (77.1%)	60 (78.9%)	14 (70%)	
**Vascular Dementia**	8 (8.3%)	6 (7.9%)	2 (10%)	0.62
**Senile Dementia**	1 (1%)	1 (1.3%)	-	
**Primary Dementia**	2 (0.9%)	2 (1.2%)	-	
**Mixed Dementia**	11 (11.5%)	7 (9.2%)	4 (20%)	
**Lewy Bodies Dementia**	-	-	-	
**Depression**	39 (17.6%)	33 (19.6%)	6 (11.3%)	0.24
**Hypertension**	136 (61.5%)	105 (62.5%)	31 (58.5%)	0.72
**T2DM ^a^**	40 (18.1%)	28 (16.7%)	12 (22.6%)	0.43
**Dyslipidemia**	86 (38.9%)	65 (38.7%)	21 (39.6%)	0.97
**Statins (*n* = 86)**	64 (74.4%)	50 (76.9%)	14 (66.7%)	0.51
**Diagnosis Time (Years)** **Depression (*n* = 38)**	14.7 (5.5)	15.3 (5.6)	11.2 (3.9)	0.09
**Diagnosis Time (Years)** **Dementia (*n* = 33)**	5.5 (3)	6 (2.9)	2.7 (1.5)	<0.05
**Difference in Diagnosis Time (Years)**	9 (4.2)	9.3 (4.4)	7.4 (2.1)	0.35

^a^ Type 2 diabetes mellitus; - No subject met that condition.

**Table 2:** Crude logistic regression (unadjusted) for dementia and AD.

	Dementia		Alzheimer’s Disease	
Variable	cOR 95% CI	*p*	cOR 95% CI	*p*
Age	1.15 (1.1–1.2)	<0.001	1.15 (1.1–1.2)	<0.001
Sex (Female)	1.4 (0.72–2.6)	0.34	1.5 (0.76–3.1)	0.23
Depression	10.4 (4.1–26.1)	<0.001	10.1 (3.9–26.2)	<0.001
Hypertension	1.25 (0.73–2.2)	0.41	1.35 (0.74–2.5)	0.33
T2DM	1.6 (0.79–3.1)	0.2	1.8 (0.87–3.7)	0.11
Dyslipidemia	0.52 (0.3–0.9)	<0.05	0.47 (0.26–0.88)	<0.05

cOR: crude Odds Ratio.

**Table 3:** Adjusted logistic regression models for dementia and AD outcome variables.

Model/Variables	Adjusted OR 95% CI	*p* Value
**Dementia**		
Model 1		
Age	1.16 (1.1–1.2)	<0.001
Depression	13.6 (4.8–38.7)	<0.001
Model 2		
Age	1.15 (1.1–1.2)	<0.001
Dyslipidemia	0.6 (0.3–1.1)	0.12 *
Model 3		
Age	1.16 (1.1–1.2)	<0.001
Depression	15.6 (5.3–45)	<0.001
Diabetes mellitus	2.6 (1.05–6.3)	<0.05
Dyslipidemia	0.54 (0.27–1.1)	0.089 **
**AD**		
Model 1		
Age	1.15 (1.1–1.2)	<0.001
Depression	11.7 (4–34.6)	<0.001
Model 2		
Age	1.15 (1.1–1.2)	<0.001
Dyslipidemia	0.53 (0.26–1.05)	0.07 ***
Model 3		
Age	1.15 (1.1–1.2)	<0.001
Depression	12.9 (4.3–39.9)	<0.001
Diabetes mellitus	2.8 (1.1–7.1)	<0.05
Dyslipidemia	0.47 (0.22–1.1)	0.056 ***

* Confounding effect was tested and did not remain in the final model. ** Confounding effector was tested and modified 12.5% of the Beta coefficient of the variable Diabetes Mellitus. *** were left in the final model due to their low *p* value.

**Table 4:** Diagnostic accuracy of logistic regression models adjusted for dementia and AD.

Outcome Variable	Model	Goodness of Fit (Nagelkerke r^2^)	Sensitivity	Specificity	Youden Index	PPV	NPV	Validity Index	AUC
**Dementia**	Model 1	0.45	71.9%	83.2%	0.55	76.7%	79.4%	78.3%	85%
	Model 2	0.32	67.7%	82.4%	0.5	74.7%	76.9%	76%	78.9%
	Model 3	0.48	77.1%	81.6%	0.59	76.3%	82.3%	79.6%	86%
**AD**	Model 1	0.43	63.5%	85.6%	0.49	72.3%	79.9%	77.4%	84.2%
	Model 2	0.32	64.9%	85.6%	0.5	72.7%	80.5%	77.9%	79.1%
	Model 3	0.48	66.2%	83.2%	0.49	70%	80.6%	76.9%	85.8%
